# Identification of the Crucial Gene in Overflow Arteriovenous Fistula by Bioinformatics Analysis

**DOI:** 10.3389/fphys.2021.621830

**Published:** 2021-08-04

**Authors:** Zhengde Zhao, Qining Fu, Liangzhu Hu, Yangdong Liu

**Affiliations:** ^1^First Affiliated Hospital of Chongqing Medical University, Chongqing, China; ^2^Department of Vascular Surgery, South China Hospital, Health Science Center, Shenzhen University, Shenzhen, China

**Keywords:** bioinformatics, overflow arteriovenous fistula, gene expression, neointimal hyperplasia, hemodialyis

## Abstract

**Objective:** The aim was to study the preliminary screening of the crucial genes in intimal hyperplasia in the venous segment of arteriovenous (AV) fistula and the underlying potential molecular mechanisms of intimal hyperplasia with bioinformatics analysis.

**Methods:** The gene expression profile data (GSE39488) was analyzed to identify differentially expressed genes (DEGs). We performed Gene Ontology and Kyoto Encyclopedia of Genes and Genomes pathway enrichment analysis of DEGs. Gene set enrichment analysis (GSEA) was used to understand the potential activated signaling pathway. The protein–protein interaction (PPI) network was constructed with the STRING database and Cytoscape software. The Venn diagram between 10 hub genes and gene sets of 4 crucial signaling pathways was used to obtain core genes and relevant potential pathways. Furthermore, GSEAs were performed to understand their biological functions.

**Results:** A total of 185 DEGs were screened in this study. The main biological function of the 111 upregulated genes in AV fistula primarily concentrated on cell proliferation and vascular remodeling, and the 74 downregulated genes in AV fistula were enriched in the biological function mainly relevant to inflammation. GSEA found four signaling pathways crucial for intimal hyperplasia, namely, MAPK, NOD-like, Cell Cycle, and TGF-beta signaling pathway. A total of 10 hub genes were identified, namely, *EGR1, EGR2, EGR3, NR4A1, NR4A2, DUSP1, CXCR4, ATF3, CCL4*, and *CYR61*. Particularly, *DUSP1* and *NR4A1* were identified as core genes that potentially participate in the MAPK signaling pathway. In AV fistula, the biological processes and pathways were primarily involved with MAPK signaling pathway and MAPK-mediated pathway with the high expression of *DUSP1* and were highly relevant to cell proliferation and inflammation with the low expression of *DUSP1*. Besides, the biological processes and pathways in AV fistula with the high expression of *NR4A1* similarly included the MAPK signaling pathway and the pathway mediated by MAPK signaling, and it was mainly involved with inflammation in AV fistula with the low expression of *NR4A1*.

**Conclusion:** We screened four potential signaling pathways relevant to intimal hyperplasia and identified 10 hub genes, including two core genes (i.e., *DUSP1* and *NR4A1*). Two core genes potentially participate in the MAPK signaling pathway and might serve as the therapeutic targets of intimal hyperplasia to prevent stenosis after AV fistula creation.

## Introduction

Arteriovenous (AV) fistula is a preferred type of vascular access for hemodialysis in patients with end-stage renal disease given its superior patency rate. However, various studies reported a poor 1-year primary patency rate of AV fistula not exceeding 60–65% (Dixon et al., 2002; Tordoir et al., 2003; Arhuidese et al., 2018). Approximately 40% of AV fistula are no longer functional 2 years after placement (Gibson et al., 2001; Arhuidese et al., 2018). The stenotic lesions in the venous outflow tract as the major late complication contribute to AV fistula failure. Percutaneous balloon angioplasty (PTA) is usually applied to maintain AV fistula patency because it reduces the need for surgical intervention, AV fistula abandonment, or AV fistula creation of more costly AV graft, given the historically greater need for maintenance intervention (Stolic, 2013). However, the restenosis after PTA occurs frequently once the first PTA is launched and that leads to repeat operative procedures until abandonment, which increases medical cost and aggravates social burden. It is relatively important to prevent stenosis after AV fistula creation and to prolong the time of primary stenosis occurrence for the management of AV fistula.

The exact pathophysiology of stenosis in AV fistula remains unclear. After AV fistula creation, the increased arterial flow alters hemodynamics state that strengthens vessel wall shear stress (Remuzzi and Ene-Iordache, 2013) and could result in a complex multidirectional and reciprocating flow in the inner side of the venous segment (Browne et al., 2015). The disturbed flow, with low and reciprocating wall shear stress, induces the selective expression of prooxidant, procoagulant, proinflammatory, and proapoptotic genes in endothelial cells (Dai et al., 2004; Chiu and Chien, 2011) and also stimulates vascular smooth muscle cell (VSMC) migration and proliferation. All the above alterations in AV fistula could result in intimal hyperplasia (Fan et al., 2010; Ene-Iordache et al., 2013). The development of intimal hyperplasia involves several vascular biological processes such as inflammation, uremia, hypoxia, shear stress, and thrombosis (Stracke et al., 2002; Roy-Chaudhury and Lee, 2007; Roy-Chaudhury et al., 2009; Wasse et al., 2012; Simone et al., 2014). However, the molecular mechanisms of intimal hyperplasia in AV fistula remain poorly understood and probably include endothelial signaling (Jones et al., 1998; Caplice et al., 2007), inflammatory and coagulation (Nath et al., 2003), extracellular matrix regulators (Misra et al., 2010), and growth factors and cell molecules (Yang et al., 2014; Sadaghianloo et al., 2017). Undoubtfully, there is an urgent need to understand the mechanism of intimal hyperplasia and find candidate target biomarkers to prevent stenosis after AV fistula creation.

The bioinformatics analysis of the microarray data is the most commonly used and successful technique to explore the pathogenesis of complicated diseases and to identify therapeutic targets. However, limited studies utilize human gene expression profiling to explore the molecular mechanism of pathophysiology in AV fistula. The microarray data of GSE39488 (Hashimoto et al., 2013) from the Gene Expression Omnibus (GEO) database, comparing the venous outflow segment of AV fistula exposed to overflow with the normal cephalic vein in hemodialysis patients, provide the opportunity for us to investigate regulatory pathways, essential genes, and their associated networks in the venous segment of AV fistula.

Therefore, we aimed to identify the crucial gene expressed in the venous outflow tract of AV fistula based on a bioinformatics approach. The exposure of the overflow of blood in AV fistula certainly and inevitably leads to intimal hyperplasia in the venous outflow tract (Hofstra et al., 1995; Tabbara et al., 2016). Thus, the identification of relevant genes might also offer better insight into the therapeutic targets of intimal hyperplasia to prevent stenosis after AV fistula creation.

## Methods

### Identification of Differentially Expressed Genes

**Figure 1:**
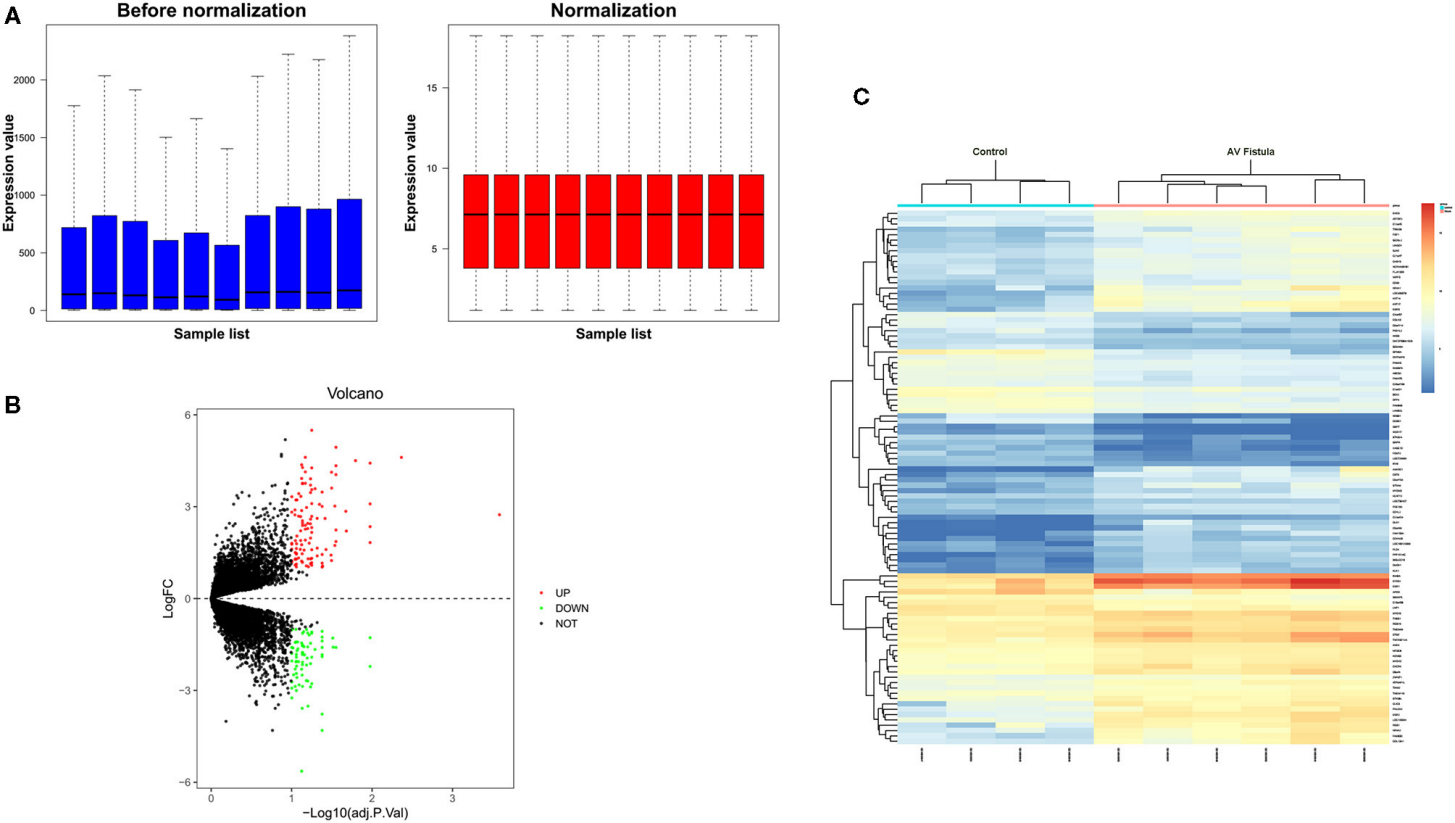
Data processing of gene expression profiles GSE39488. **(A)** Normalization of gene expression profiling data with quantile method. The blue plot represents the data before normalization, and the red plot represents the data after normalization. **(B)** Volcano plot of differentially expressed genes. The red dots represent the upregulated genes in the AV fistula group compared with the control group (log_2_FC > 1 and < *P* < 0.1); the green dots represent the downregulated genes in the AV fistula group compared with the control group (log_2_FC < −1 and *P* < 0.1); and the black spots represent genes without significant difference (|log_2_FC| <1 and *P* > 0.1). **(C)** Cluster heatmap of the top 100 differentially expressed genes (ranked by log_2_FC); blue indicates a relatively low expression (log_2_FC > 1), and red indicates a relatively high expression (log_2_FC < −1). Each column represents a sample, and each row represents a gene. Samples show comparable gene expression patterns within a group but show divergent gene expression patterns between groups. FC, fold change.

The GSE39488 dataset was obtained from the public GEO database from the American National Center for Biotechnology Information (www.ncbi.nlm.nih.gov/geo). The microarray data of GSE39488 based on the platform of GPL10332 (Agilent-026652 Whole Human Genome Microarray 4x44K v2) contain 10 samples, including six samples collected from the venous segments of AV fistula in hemodialysis patients with overflow in AV fistula and four control samples collected from normal cephalic veins in hemodialysis patients (Table 1). Normalization of gene expression profiling data with quantile method, the result showed in Figure 1A. To identify the differentially expressed genes (DEGs) between the AV fistula group and the control group, the “LIMMA” package in R software (version 3.6.1; https://www.rproject.org/) was used to calculate the *P*-value and false discover rate (FDR). The criteria of statistically significant DEGs were set as follows: *P* < 0.1 (adjusted with FDR) and |log_2_ fold change (FC)| > 1. The genes with log_2_ FC > 1 and with log_2_ FC < −1 were defined as upregulated DEGs and downregulated DEGs, respectively. A volcano plot was mapped to present all of the DEGs using “ggplot2” packages. The heatmap for the gene expression data of the top 100 DEGs (ranked by adjusted *P*-value) was performed with “Heatmap” packages.

**Table 1:** Information of samples in GSE39488 from the Gene Expression Omnibus (GEO) database.

**Group**	**Samples**	**Platform**	**Title**	**Gender**	**Tissue**	**Disease sate**
AV fistula	GSM969946	GPL10332	AV fistula patient 1	Male	Venous segment of AV fistula	Overflow AV fistula
	GSM969949	GPL10332	AV fistula patient 2	Male	Venous segment of AV fistula	Overflow AV fistula
	GSM969950	GPL10332	AV fistula patient 3	Male	Venous segment of AV fistula	Overflow AV fistula
	GSM969951	GPL10332	AV fistula patient 4	Male	Venous segment of AV fistula	Overflow AV fistula
	GSM969952	GPL10332	AV fistula patient 5	Female	Venous segment of AV fistula	Overflow AV fistula
	GSM969953	GPL10332	AV fistula patient 6	Female	Venous segment of AV fistula	Overflow AV fistula
Control	GSM969945	GPL10332	Control patient 1	Female	Normal cephalic vein	Control
	GSM969954	GPL10332	Control patient 4	Male	Normal cephalic vein	Control
	GSM969947	GPL10332	Control patient 2	Female	Normal cephalic vein	Control
	GSM969948	GPL10332	Control patient 3	Male	Normal cephalic vein	Control

*AV fistula, arteriovenous fistula*.

### Enrichment Analysis of Differentially Expressed Genes

To understand the biological function of DEGs in the venous segment of AV fistula, Gene Ontology (GO) and Kyoto Encyclopedia of Genes and Genomes (KEGG) pathway enrichment of DEGs were all performed by the well-known Database for Annotation, Visualization, and Integrated Discovery (DAVID) (version 6.8, https://david.ncifcrf.gov/) online bioinformatics tools (Udhaya Kumar et al., 2020). DAVID is a significant source for any functional evaluation of the high-throughput gene expression profiles. The GO enrichment analysis consisted of three categories, namely, cellular component, molecular function, and biological process. The *P* < 0.05 was the criteria for statistically significant enriched GO term and KEGG pathways, and the top five significant terms were visualized using “ggpubr” package in R software.

### Gene Set Enrichment Analysis

Gene set enrichment analysis (GSEA) is a knowledge-based powerful computational analytical method for interpreting genome-wide expression profiles to determine whether *a priori* defined set of genes shows statistical, significant, and concordant differences between two biological states. In this study, to further evaluate the performance of the Fisher's score algorithm in selecting feature genes, the gene expression data of all annotated 22,457 genes from GSE39488 were submitted into GSEA Desktop version 4.0.3 with GSEAPreranked mode (Subramanian et al., 2005) based on the gene set c2 (cp.kegg.v.6.2.symbols.gmt) for signaling pathway and c5 (bp.v.6.2.symbols.gmt) for biological process. All the samples were divided into AV fistula group and control group. The number and type of permutations were set at “1,000” and “gene set,” respectively. The terms of the enriched pathways were arranged in the order of their normalized enrichment scores (NESs). The cutoff criteria of statistically significant enriched pathways were defined as follows: the absolute value of NES > 1, nominal *P* < 0.05, and FDR <0.25.

### Protein–Protein Interaction Networks

We submitted the DEGs into the online search tool in the STRING database (version 11.0, http://string-db.org) to create the protein–protein interaction (PPI) networks. The confident score ≥0.4 (i.e., medium confidence) was set as the cutoff to obtain significant PPI networks. The significant interactions were visualized in Cytoscape software (version 3.7.2, https://www.cytoscape.org), and the network was plotted for both upregulated and downregulated DEGs in the AV fistula group compared with the control group, and the size of nodes labeled the Maximal Clique Centrality (MCC) value of each gene. The cytoHubba plugin in Cytoscape software provides a user-friendly interface to explore important nodes in biological networks. It provides 11 topological algorithms for identifying the complex network of hub genes. Among all the algorithms, MCC has a better performance in predicting the PPI network of hub genes (Chin et al., 2014). The MCC algorithm was employed to identify the hub genes in this study.

## Results

### Identification of Significant Differentially Expressed Genes

In this study, we screened 185 DEGs containing 111 upregulated genes and 74 downregulated genes in the venous tissue of AV fistulas exposed to high blood flow compared with the normal cephalic vein in hemodialysis patients. Volcano plots (Figure 1B) and heatmap (Figure 1C) were drawn to visualize the screened DEGs.

### Gene Ontology and KEGG Enrichment Analysis of Differentially Expressed Genes

**Figure 2:**
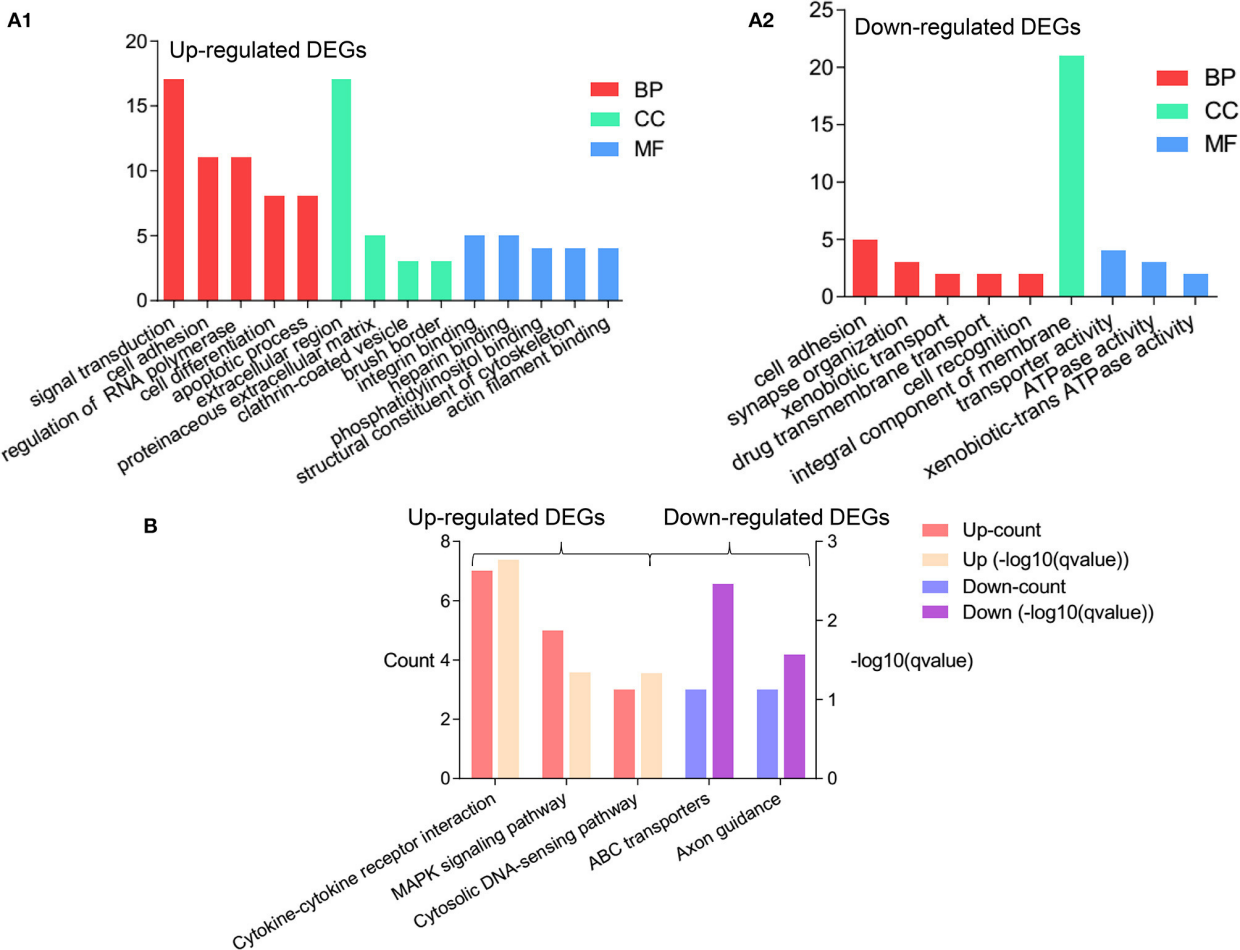
The significant results of enrichment analysis from the Database for Annotation, Visualization, and Integrated Discovery (DAVID) website. The cutoff criterion was *P* < 0.05. **(A1)** The GO functional enrichment analysis of the upregulated DEGs (log_2_FC > 1) based on the three types of sub-ontologies (Biological Processes: top two terms; Molecular Function: four terms; Cellular Component: top five terms); ordinate axis represents the number of DEGs under the GO term. **(A2)** The GO functional enrichment analysis of the upregulated DEGs (log_2_FC > 1) based on the three types of sub-ontologies (Biological Processes: top five terms; Molecular Function: one term; Cellular Component: three terms); ordinate axis represents the number of DEGs under the GO term. **(B)** The enrichment of the KEGG pathway analysis of the upregulated DEGs (left) in the AV fistula group compared with the control group and the downregulated DEGs (right) in the AV fistula group compared with the control group; ordinate axis at left represents the count of DEGs under the KEGG pathway term and ordinate axis at right represents the *q*-value of DEGs under the KEGG pathway term. DEGs, differentially expressed genes; GO, Gene Ontology.

The DAVID database was used for the GO and KEGG analysis of 111 upregulated DEGs and 74 downregulated DEGs in the AV fistula group compared with the control group (significance threshold, *P* < 0.05), respectively. For the upregulated genes in the AV fistula group compared with the control group, the top five enriched GO terms were “skeletal muscle cell differentiation,” “cell adhesion,” “signal transduction,” “positive regulation of natural killer cell chemotaxis,” and “angiogenesis” in Biological Processes (BP) category, and “extracellular region,” “brush border,” “clathrin-coated vesicle,” and “proteinaceous extracellular matrix” in Cellular Component (CC) category, and “CCR1 chemokine receptor binding,” “CCR5 chemokine receptor binding,” “integrin binding,” “heparin binding,” and “phosphatidylinositol binding” in Molecular Function (MF) category. The results are presented in Figure 2A1 and Supplementary Table 1. For the downregulated genes in the AV fistula group compared with the control group, the main enriched GO terms were “synapse organization,” “xenobiotic transport,” “cell adhesion,” “drug transmembrane transport,” and “cell recognition” in BP category, only “integral component of membrane” in CC category, and “ATPase activity,” “coupled to transmembrane movement of substances,” “xenobiotic-transporting ATPase activity,” and “transporter activity” in MF category. The results are presented in Figure 2A2 and Supplementary Table 2. As for the KEGG pathway, the upregulated genes in the AV fistula group compared with the control group were significantly enriched in “cytokine–cytokine receptor interaction,” “MAPK signaling pathway,” and “cytosolic DNA-sensing pathway,” and the downregulated genes in the AV fistula group compared with the control group were significantly enriched in “ATP binding cassette (ABC) transporters” and “Axon guidance.” The results are presented in Figure 2B and Supplementary Table 3.

### Gene Set Enrichment Analysis

**Figure 3:**
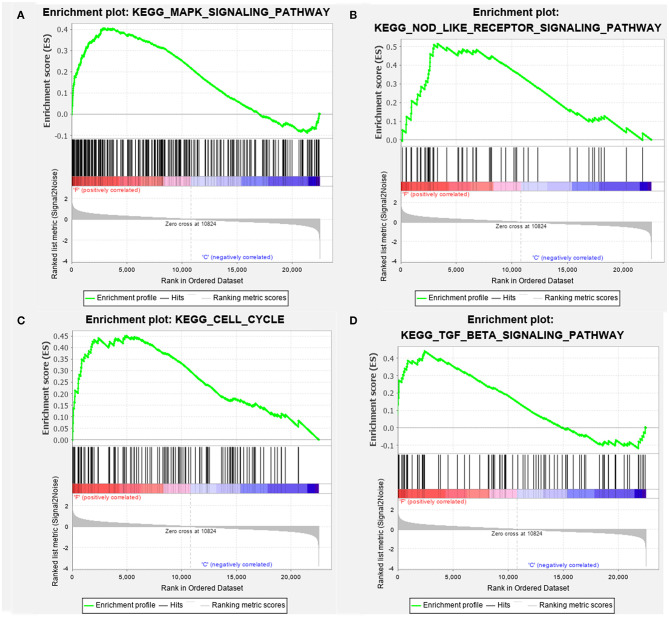
The enriched signaling pathway through the gene set enrichment analysis (GSEA). **(A)** MAPK signaling pathway, **(B)** NOD-like signaling pathway, **(C)** Cell Cycle, and **(D)** TGF-beta signaling pathway. The gene set c2 (cp.kegg.v.6.2.symbols.gmt) database was used to analyze the whole gene expression value of the AV fistula and control samples. The cutoff criteria of the significant gene set were the absolute value of normalized enrichment score (NES) > 1, nominal *P* < 0.05, and FDR <0.25. The enrichment plots **(A–D)** illustrate the high expression of the four gens set in the AV fistula group. F, AV fistula; C, Control.

For the GSEA of the pathways, the gene sets of the “MAPK signaling pathway,” “NOD-like signaling pathway,” “Cell Cycle,” and “TGF-beta signaling pathway” were significantly enriched in overflow AV fistula (Figure 3). The results of GSEA of KEGG pathways were presented in Table 2 (Detailed in Supplementary Table 4).

**Table 2:** The significantly enriched KEGG pathway from GSEA.

**Pathway**	**SIZE**	**ES**	**NES**	**NOM *P*-value**	**FDR *q*-value**
Overflow arteriovenous fistula^*^					
KEGG_leishmania_infection	67	0.55	2.24	0.000	0.000
KEGG_small_cell_lung_cancer	84	0.50	2.14	0.000	0.002
KEGG_focal_adhesion	194	0.43	2.10	0.000	0.002
KEGG_MAPK_signaling_pathway	261	0.40	2.05	0.000	0.002
KEGG_cell_cycle	116	0.45	2.04	0.000	0.001
KEGG_NOD_like_receptor_signaling_pathway	56	0.51	2.03	0.000	0.002
kEGG_regulation_of_actin_cytoskeleton	209	0.41	2.01	0.000	0.003
KEGG_bladder_cancer	39	0.54	1.97	0.000	0.004
KEGG_chronic_myeloid_leukemia	73	0.48	1.95	0.000	0.004
KEGG_p53_signaling_pathway	63	0.48	1.92	0.000	0.006
KEGG_TGF_beta_signaling_pathway	85	0.44	1.90	0.000	0.007
KEGG_pathogenic_escherichia_coli_infection	50	0.48	1.89	0.000	0.007
KEGG_leukocyte_transendothelial_migration	114	0.41	1.86	0.000	0.009
KEGG_chemokine_signaling_pathway	178	0.38	1.85	0.000	0.010
KEGG_pancreatic_cancer	68	0.45	1.84	0.000	0.011
Normal cephalic vein					
KEGG_ribosome	86	0.56	2.40	0.000	0.000
KEGG_ABC_transporters	44	0.61	2.31	0.000	0.000
KEGG_renin_angiotensin_system	16	0.66	1.91	0.000	0.013
KEGG_fatty_acid_metabolism	39	0.49	1.78	0.000	0.036
KEGG_propanoate_metabolism	31	0.51	1.78	0.000	0.029
KEGG_histidine_metabolism	27	0.52	1.76	0.000	0.030
KEGG_metabolism_of_xenobiotics_by_cytochrome_p450	59	0.43	1.72	0.000	0.036
KEGG_glycerolipid_metabolism	43	0.43	1.62	0.015	0.081
KEGG_drug_metabolism_cytochrome_p450	60	0.40	1.62	0.006	0.073
KEGG_butanoate_metabolism	33	0.46	1.61	0.014	0.066
KEGG_nitrogen_metabolism	23	0.50	1.60	0.021	0.067
KEGG_peroxisome	75	0.38	1.59	0.004	0.068
KEGG_PPAR_signaling_pathway	67	0.37	1.54	0.016	0.095
KEGG_tyrosine_metabolism	40	0.41	1.47	0.037	0.149

* *Only top 15 significantly enriched KEGG pathways were presented*.

### Protein–Protein Interaction Networks

**Figure 4:**
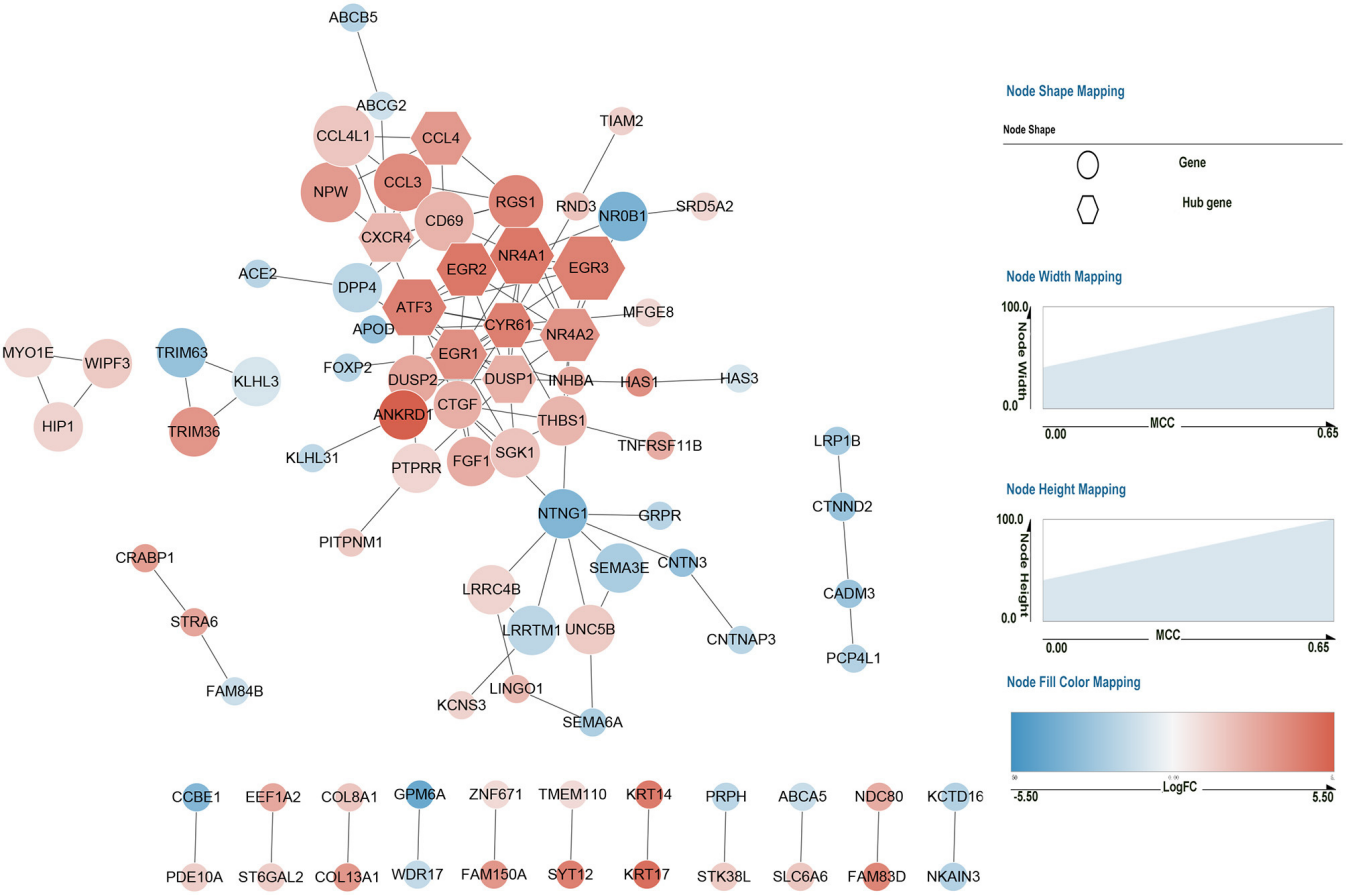
Protein–protein interaction network of 185 DEGs. Red nodes represent the upregulated genes (log_2_FC > 1) in the AV fistula group compared with the control group, and blue nodes represent the downregulated genes (log_2_FC < −1) in the AV fistula group compared with the control group. The nodes represent proteins. Each line between nodes represents the interaction relationship between two nodes. The size (i.e., width and height) of each node represents the rank score calculated by the MCC method in Cytoscape software. The color depth represents the value of log_2_FC; the bluer the color, the lower the log_2_FC value; and the redder the color, the larger the log_2_FC value. The hexagonal nodes represent the identified hub gene, and the circular nodes represent the DEGs except the hub genes. FC, fold change; MCC, Maximal Clique Centrality.

We submitted all the 185 DEGs to the online search tool in the STRING database and constructed a PPI network that consisted of 85 nodes and 119 edges in Cytoscape software. The network is displayed in Figure 4. To identify the key gene leading to cascade the alteration of gene expression in AV fistula, the rank score of each gene was calculated in cytoHubba, and the top 10 hub genes were selected based on the MCC algorithm as follows: *EGR1, NR4A1, ATF3, NR4A2, EGR2, DUSP1, EGR3, CXCR4, CCL4*, and *CYR61*.

### Further Bioinformatics Analysis for DUSP1 and NR4A1

The Venn diagram between 10 hub genes and gene set of the MAPK signaling pathway, NOD-like signaling pathway, Cell Cycle, and TGF-beta signaling pathway showed *DUSP1* and *NR4A1* that belong to the gene set of MAPK signaling pathway (Gene set information in Supplementary Table 5). For further analysis of *DUSP1* and *NR4A1*, the six samples of venous tissue from overflow AV fistula were divided into high-expression groups and low-expression groups according to the median expression value of the *DUSP1* and *NR4A1* gene. The GSEA was performed to compare the biological processes and pathways between the high-expression group and the low-expression group.

**Figure 5:**
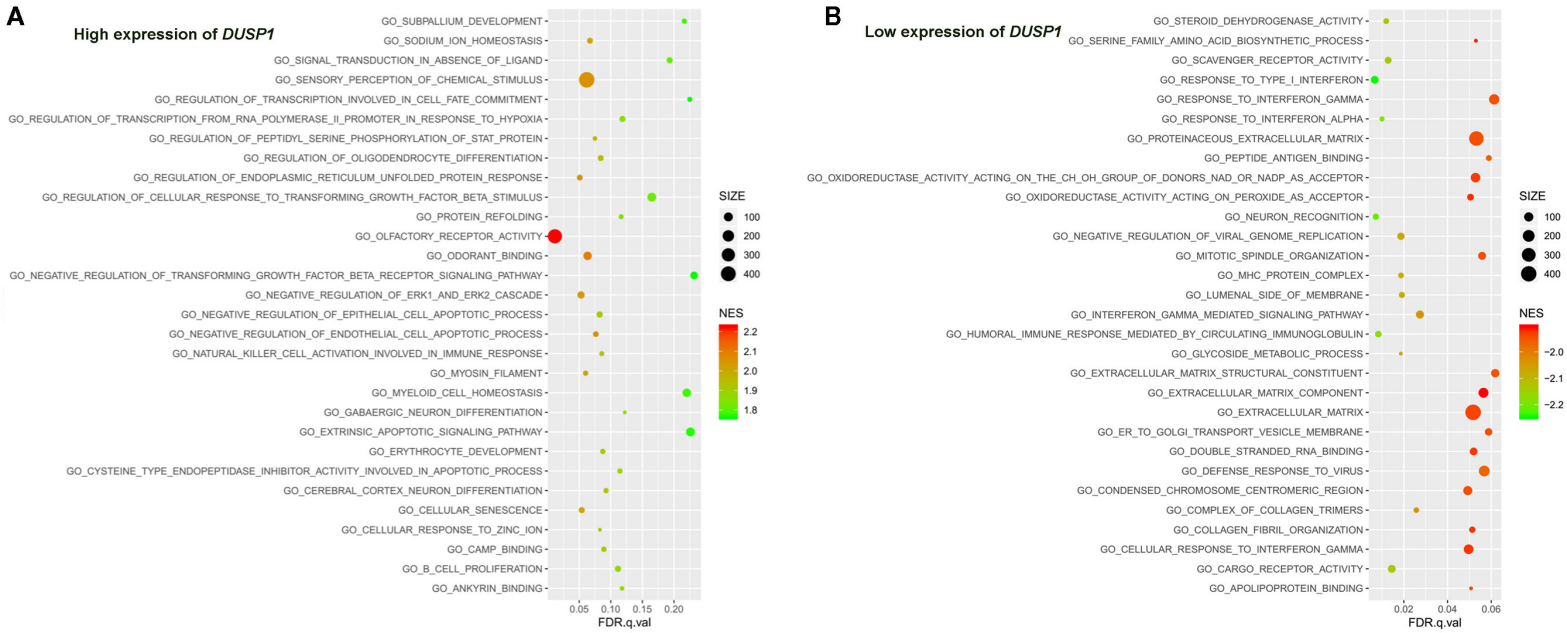
The results of the GSEA on the biological processes. **(A)** Biological processes (Top 30) in the *DUSP1* high-expression group and **(B)** biological processes (Top 30) in the *DUSP1* low-expression group. The size of the node represents the number of genes under the gene set. The color of the node represents the NES of the gene set. The horizontal axis represents the level of *q*-value.

**Figure 6:**
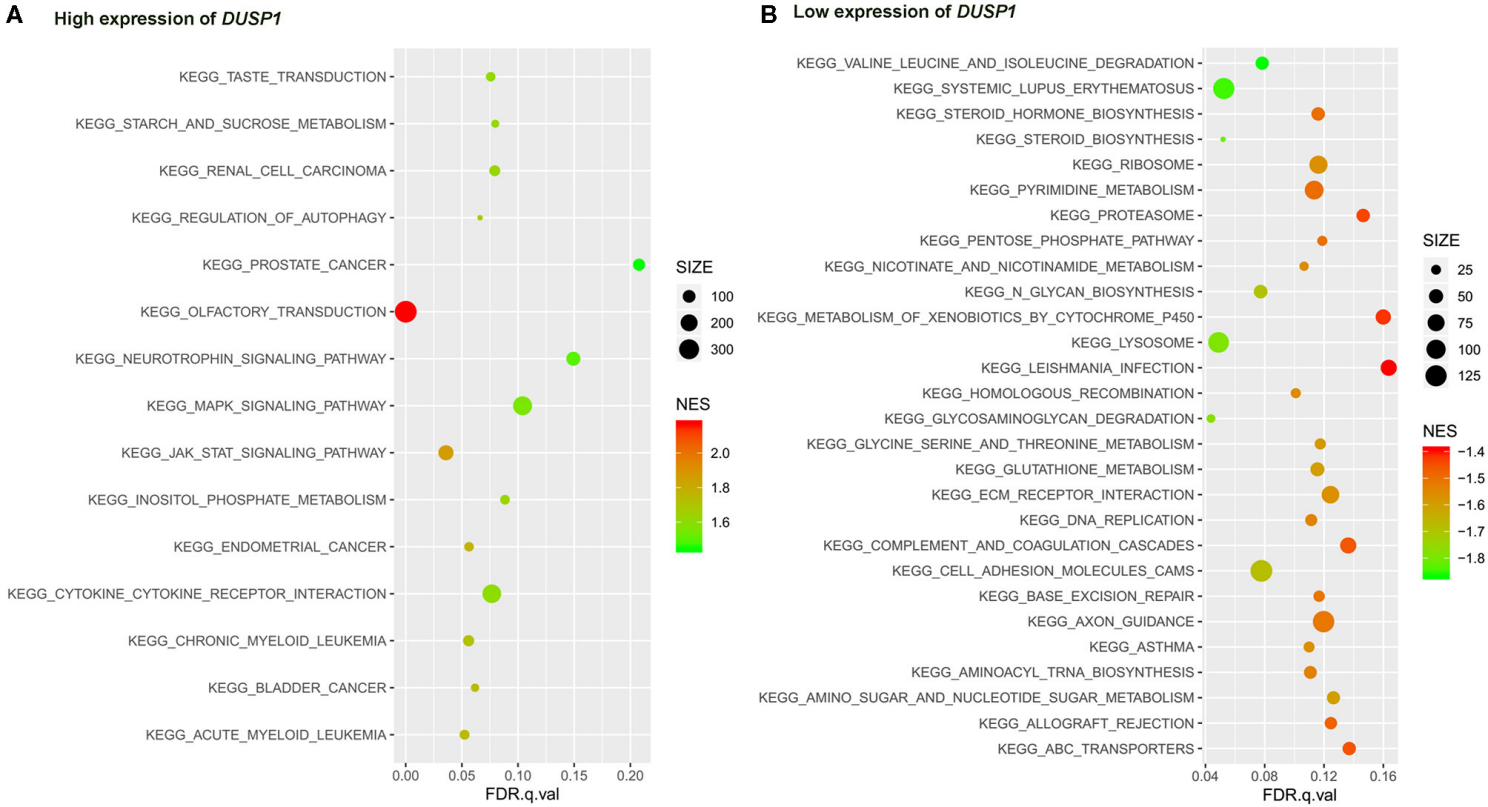
The results of the GSEA on the KEGG pathway. **(A)** KEGG pathway in the high expression of *DUSP1* in AV fistula and **(B)** KEGG pathway in the low expression of *DUSP1* in AV fistula. The size of the node represents the number of genes under the gene set. The color of the node represents the NES of the gene set. The horizontal axis represents the level of *q*-value.

The primary gene sets of biological processes and pathways in AV fistula with the high expression of *DUSP1* included MAPK signaling pathway, TGF-beta receptor signaling, responses to TGF-beta signaling, regulation of ERK1 and ERK2 cascade, and JAK/STAT signaling. The gene sets of biological processes and pathways in AV fistula with the low expression of *DUSP1* were majorly relevant to cell proliferation such as DNA replication, mitotic nuclear division, and inflammation containing the response to interferon-γ, immunity, ABC transporters, and cell adhesion molecules (Figures 5, 6; Supplementary Tables 6, 7).

**Figure 7:**
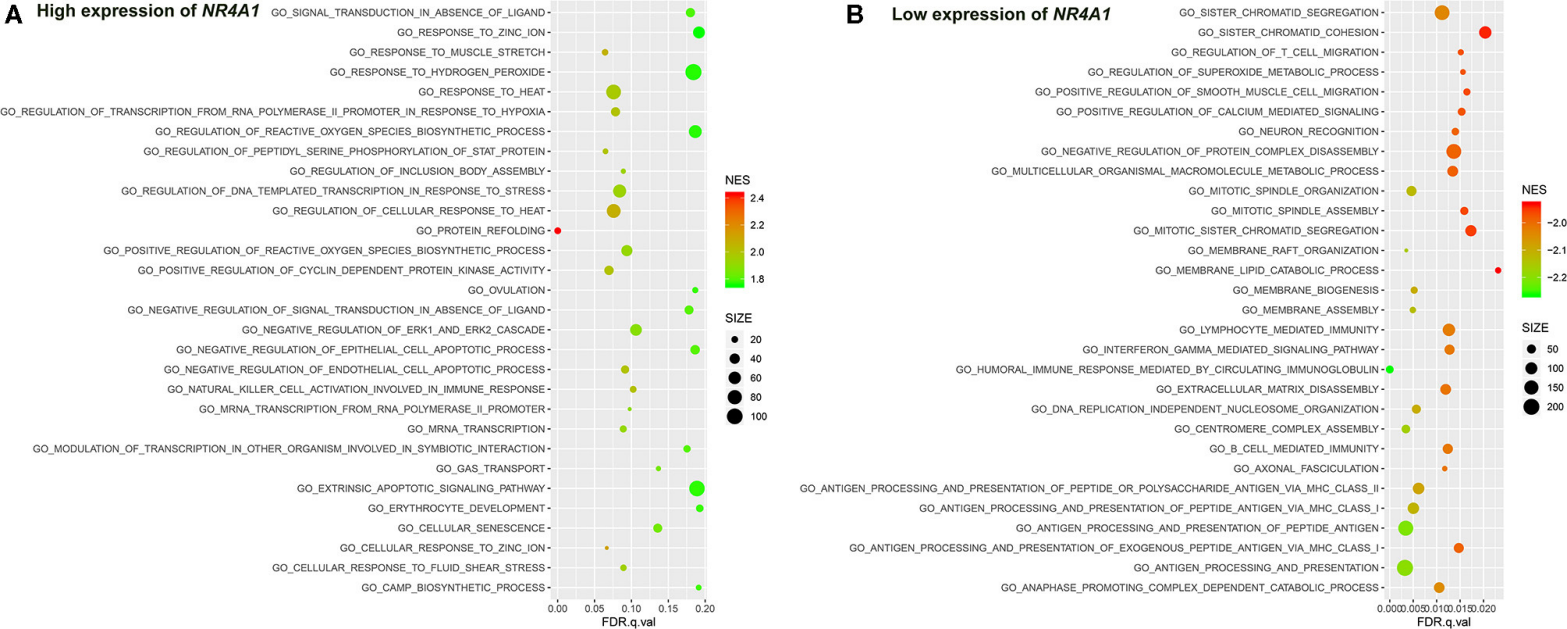
The results of the GSEA on the biological processes. **(A)** Biological processes (Top 30) in the high expression of *NR4A1* in AV fistula and **(B)** biological processes (Top30) in the low expression of *NR4A1* in AV fistula. The size of the node represents the number of genes under the gene set. The color of the node represents the NES of the gene set. The horizontal axis represents the level of *q*-value.

**Figure 8:**
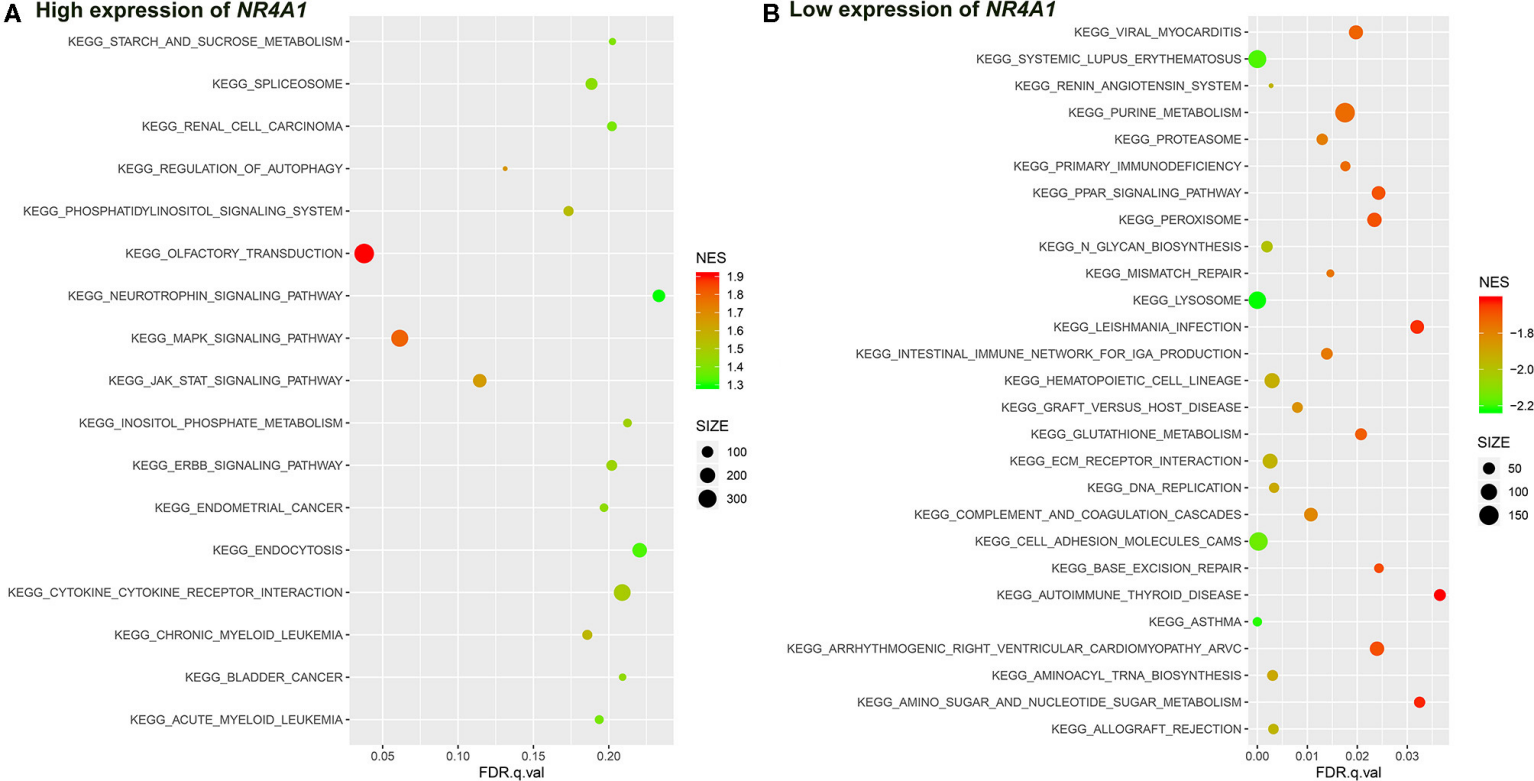
The results of the GSEA on the KEGG pathway. **(A)** KEGG pathway in the high expression of *NR4A1* in AV fistula and **(B)** KEGG pathway in the low expression of *NR4A1* in AV fistula. The size of the node represents the number of genes under the gene set. The color of the node represents the NES of the gene set. The horizontal axis represents the level of *q*-value.

We identified several gene sets of biological processes and pathways in patients with the high expression of *NR4A1* in AV fistula, which includes MAPK signaling and TGF-beta signaling, regulation of ERK1 and ERK2 cascade, ErbB signaling, and JAK/STAT signaling. The biological processes and pathways in patients with the low expression of *NR4A1* were mainly involved with inflammation such as cell adhesion, ECM–receptor interaction, and immunity responses (Figures 7, 8; Supplementary Tables 8, 9).

## Discussion

Although AV fistula is the recommended option for hemodialysis patients, stenosis due to intimal hyperplasia is the main obstacle for maintaining AV fistula patency. Thus, the analysis of gene expression of overflow AV fistula could provide insight into the molecular mechanism of intimal hyperplasia.

Microarray technology is one of the important ways to explore gene expression related to complex disorders and could help us to predict target genes for treatment. In this study, we screened 185 DEGs containing 111 upregulated genes and 74 downregulated genes in the AV fistula group compared with the control group. These identified DEGs were subjected to the GO and KEGG enrichment analysis to acknowledge the potential biological process and signaling pathway. GSEA was used to explore the potentially activated biological processes and signaling pathways between the AV fistula group and the control group with the advantage of avoiding undetectable, small changes in the expression of single genes (Mootha et al., 2003). Subsequently, we subjected the identified DEGs to the STRING database, which exploits the complex interactions between DEGs based on evidence from experiments and repositories. In the PPI network, the nodes represented proteins, the edges between nodes represented their interactions, and the top 10 genes ranked by MCC indicating high weight between DEGs in the network were filtered as hub genes that might be important in the regulation of biological activity in AV fistula. The gene set of a signaling pathway to which a gene belongs suggests that it potentially participates in the pathway. The intersection of hub gene and four crucial gene set of signaling pathway help us to determine in which pathway the hub gene participated. Our study identified two hub genes as core genes that potentially participated in the MAPK signaling pathway.

The GO and KEGG enrichment helped us to explore the involvement of the 185 identified DEGs in BP, MF, CC, and pathways and to determine the functional annotation of these genes. As shown in Supplementary Tables 1, 2, the GO enrichment of upregulated genes in the venous segment of AV fistula was mainly involved with cell adhesion, intracellular signal transduction, and angiogenesis, while the downregulated genes were primarily enriched in the biological function relevant to apoptosis (Kong et al., 2001; Schumacher et al., 2010; Poon et al., 2014; Barth et al., 2017). Those results were in line with the previous report and crucial for intimal hyperplasia. After AV fistula creation, the hemodynamics change in outflow vein results in the short-term and long-term vascular remodeling (Remuzzi and Ene-Iordache, 2013), and sustained expression of several molecules that cause the activation of a series of pathways stimulating intimal hyperplasia (Chiu and Chien, 2011; Zafrani and Ince, 2015). Shear stress can improve the expression of surface adhesion molecules (i.e., vascular cell adhesion protein 1) (Chiu and Chien, 2011). Mechanical injury in the artery could activate a mild adventitial angiogenic response and aggravate neointima formation (Chiu and Chien, 2011). Neoangiogenesis has also been observed in the AV fistula rat model (Chiu and Chien, 2011). The activation of apoptosis in VSMCs can reduce intimal hyperplasia (Aizawa et al., 2001).

The KEGG enrichment analysis screened a total of five significant pathways based on the DEGs. Simultaneously, GSEA identified 46 significant gene set/pathways in AV fistula and 14 significant gene set/pathways in normal cephalic vein. All those significant pathways in AV fistula were concentrated on proliferation, apoptosis, cytokine–cytokine interaction, intimal hyperplasia, inflammation response, and cancer-related pathways. Interestingly, MAPK signaling pathway, NOD-like signaling pathway, Cell Cycle, and TGF-beta signaling pathway have been reported to play a prominent role in the development of intimal hyperplasia. The dysregulation of the MAPK signaling pathway leads to uncontrolled cell proliferation, migration, and differentiation. Recent studies reported that the MAPK signaling pathway is associated with intimal hyperplasia by the ERK1/2 signaling pathway (Qu et al., 2020). The inhibition of the MAPK pathway decreases the thickening of the intima and limits vascular remodeling (Ge et al., 2013; Yang et al., 2020). NOD-like receptor signaling pathway could trigger a family of pattern recognition receptors that mediate the initial innate immune response to cellular injury and stress. The activated signal molecule of NOD-like receptor family such as NLRP3 inflammasome could be aggregated to form an inflammasome complex and to enhance neointima formation (Xia et al., 2014; Koka et al., 2017). Cell cycle regulatory factors could promote VSMC migration and proliferation in the pathogenesis of intimal hyperplasia (Wei et al., 1997; Zuckerbraun et al., 2003). A study that detected the cell cycle regulators in 18 failed human AV fistula demonstrated that the regulators positively regulating the cell cycle progression were significantly higher in AV fistula than normal vein and arteries (de Graaf et al., 2003). TGF-beta is a family of regulatory proteins containing many multifunctional cytokines, such as TGF-beta, bone morphogenetic proteins (BMPs), activins, inhibins, and mycostatin (Bobik, 2006). The regulatory proteins of TGF-beta family participate in a wide range of biological processes such as cell growth and differentiation (ten Dijke et al., 1996). The expression of TGF-beta in neointimal and medial layers is higher in stenosed hemodialysis fistula (Stracke et al., 2002). The previous study has revealed that the overexpression of TGF-beta stimulates intimal hyperplasia and extracellular matrix accumulation (Nabel et al., 1993). Additionally, targeting the TGF-beta mRNA degradation attenuates neointima growth (Yamamoto et al., 2000).

From the analysis of the PPI network, 10 DEGs with the highest MCC score were filtered as hub genes, namely, *EGR1, EGR2, EGR3, NR4A1, NR4A2, ATF3, CXCR4, CCL4, CYR61*, and *DUSP1*. As shown in Supplementary Figure 2, the expression level of the 10 hub genes was notably increased in the patients with AV fistula compared with the normal controls. Early growth response genes are the family of zinc-finger transcription factors comprising of four members, namely, *EGR1, EGR2, EGR3*, and *EGR4*. They are expressed in many different cell types and could be rapidly induced in response to mitogens, differentiation, apoptotic signals, and tissue injury (Decker et al., 1998). *EGR1* is crucial for intimal hyperplasia and vascular remodeling after vascular injury (Huang et al., 2010), balloon injury (Han and Liu, 2010), arterial hypertension (van der Feen et al., 2016), and chemical damage (Vazquez-Padron et al., 2010). The inhibition of *EGR1* expression attenuates intimal hyperplasia through the downregulation of TGF-beta and the upregulation of nitric oxide synthase (NOS) (Liu et al., 2008; Han and Liu, 2010). In our study, an augmented expression level of *EGR1* might enhance intimal hyperplasia in the venous segment of AV fistula exposed to high blood flow, indicating *EGR1* could be one novel regulatory biomarker in AV fistula. *EGR2* and *EGR3* were essential for the regulation of immune response and the development of inflammation (Li et al., 2012; Sumitomo et al., 2013) but have not been proved to be associated with intimal hyperplasia especially in AV fistula.

The *NR4A* subfamilies of nuclear receptors such as *NR4A1* and *NR4A2* become significant regulators in the developing neointima and play a critical role in several aspects of vascular remodeling such as cell viability, proliferation, and inflammation (Zhao and Bruemmer, 2010). Some studies indicated that the activation of *NR4A1* and *NR4A2* could inhibit VSMC proliferation and protect against neointima formation (Pires et al., 2007; Bonta et al., 2010). But Liu et al. found that *Nur77*(*NR4A1*)-targeted siRNA could mitigate autologous vein graft stenosis (Liu et al., 2015). In our study, the expression level of *NR4A1* and *NR4A2* in the AV fistula group is higher than that in the control group, which were consistent with the result in the autologous vein graft stenosis model (Liu et al., 2015) but not in the cuff-induced neointima formation model (Pires et al., 2007) and the carotid artery ligation-induced neointima model (Bonta et al., 2010). That is because the hemodynamics state in AV fistula might be similar to the autologous vein graft stenosis model.

*CYR61* plays a role in cell proliferation, differentiation, angiogenesis, apoptosis, and extracellular matrix formation. The knockdown of *CYR61* suppresses intimal hyperplasia in a rat artery balloon injury model and regulates the proliferation of VSMCs (Lee et al., 2007; Matsumae et al., 2008). The expression of *CYR61* increased in our study, which might enhance cell proliferation and intimal hyperplasia in AV fistula.

CXCL12/CXCR4 chemokine ligand/receptor axis mediates the mobilization of smooth muscle cell (SMC) progenitors, driving injury-induced neointimal hyperplasia. Endothelial *CXCR4* deficiency significantly increased wire injury-induced neointima formation in carotid arteries (Noels et al., 2014). The inhibition of *CXCR4* could effectively prevent neointima formation (Hamesch et al., 2012). Our dataset showed an augmented expression of *CXCR4*, which might activate CXCL12/CXCR4 chemokine ligand and subsequently result in neointimal hyperplasia in AV fistula. The inhibition of *CCL4* could attenuate the expression of E-selectin, vascular cell adhesion molecule-1 (VCAM-1), and intercellular adhesion molecule-1 (ICAM-1) (Chang et al., 2020) and further attenuate intimal hyperplasia and reduce inflammation (Yasukawa et al., 1997; Gotoh et al., 2004). Additionally, the G-protein-coupled receptor (CCR5) is activated by the inflammatory chemokines CCL3, CCL4, and CCL5, but only CCL4 exhibits selectivity for CCR5, which stimulates intimal hyperplasia in vessels (Maguire et al., 2014). Therefore, the high expression of *CCL4* in AV fistula might potentially activate CCR5 or promote the expression of E-selectin, VCAM-1, and ICAM-1 and subsequently contributes to intimal hyperplasia. The activation of *ATF3*, which is involved in the complex process of the cellular stress response, is a pivotal target of neointima formation and VSMC proliferation in response to wire injury and balloon injury in vascular cells (Huang et al., 2014).

*DUSP1* is a dual-specific phosphatase that regulates the MAPK pathway which controlled a vast of cellular processes. The endothelial exposure of high shear stress induces the persistent expression of *DUSP1* and mitigates the activities of p38 MAPK and JNK pathway (Zakkar et al., 2008). Recent studies have revealed that the attenuation of ERK induced by *DUSP1* inhibits intimal hyperplasia by reducing proliferation and inducing apoptosis of SMCs (Kim et al., 2011).

As mentioned earlier, each of 10 hub genes (*EGR1, NR4A1, ATF3, NR4A2, EGR2, DUSP1, EGR3, CXCR4, CCL4*, and *CYR61*) plays a very important role in intimal hyperplasia which is the culprit of AV fistula stenosis causing the loss of hemodialysis access in the patients with end-stage renal disease. Although the molecular mechanisms and pathways between 10 hub genes and intimal hyperplasia are diverse, those 10 genes might provide us various effective targets to suppress intimal hyperplasia with the design of gene-therapeutic drug-eluted balloons or screening specific medicine to prevent AV fistula stenosis in the future study.

Furthermore, GSEA showed that the biological processes and pathways in AV fistula with the high expression of *DUSP1* were primarily involved with MAPK signaling pathway, i.e., MAPK signaling-mediated pathway such as TGF-beta receptor signaling, regulation of ERK1 and ERK2 cascade, and JAK/STAT signaling (Meyer and Levine, 2014; Zhang et al., 2014; Li et al., 2019; Hawke et al., 2020), and others involved with cell apoptosis. However, the biological processes and pathways in AV fistula with the low expression of *DUSP1* were majorly relevant to cell proliferation such as DNA replication, mitotic nuclear division, and inflammation containing the response to interferon-γ, immunity, ABC transporters, and cell adhesion. As discussed previously, *DUSP1* mediated the phosphorylation process in negative regulation of MAPK signaling pathway and ERK cascade such as ERK1 and ERK2 as the downstream of MAPK signaling pathway (Abraham and Clark, 2006). TGF-beta has been shown to induce the expression of *DUSP1*, which leads to the inactivation of JNK or p38 MAPK signaling pathway (Xiao et al., 2002; Jono et al., 2003; Mikami et al., 2006). The inhibition of MAPK signaling pathway could suppress cell proliferation and intimal hyperplasia (Ge et al., 2013; Qu et al., 2020; Yang et al., 2020). In our study, the biological processes and pathways in AV fistula with the low expression of *DUSP1* were partly concentrated on cell proliferation, indicating the probable promotion of cell proliferation by the p38 MAPK pathway. Based on the data mentioned above, we can speculate that *DUSP1* is a crucial gene in AV fistula and potentially participates in cell proliferation and intimal hyperplasia.

*NR4A1* also belongs to the MAPK signaling pathway. GSEA screened several biological processes and pathways in patients with the high expression of *NR4A1* in AV fistula, such as MAPK signaling and TGF-beta signaling, ErbB signaling, regulation of ERK1 and ERK2 cascade, all of which are mediated by MAPK signaling pathways (Nie and Chang, 2007; Li et al., 2019; Hawke et al., 2020). JAK-mediated signaling may partly rely on the activation of the MAPK signaling axes (Meyer and Levine, 2014). But the biological processes and pathways in patients with the low expression of *NR4A1* were mainly involved with inflammation such as cell adhesion, ECM–receptor interaction, and immune network. The role of *NR4A1* in the regulation of vascular remodeling by the MAPK signaling pathway remains unknown. ERK1/2 as the downstream of MAPK signaling pathway could upregulate *NR4A1* expression, and MAPK phosphatase-1 could negatively regulate the induction of *NR4A1* dependent on ERK1/2 (Mori Sequeiros Garcia et al., 2015). In macrophages, the p38 MAPK signaling pathway could promote the expression of *NR4A1* and subsequently protects against inflammation (Shao et al., 2010). The activation of *NR4A1* could diminish the NF-κB activation partly dependent on p38 MAPK activity. Furthermore, the activation of NF-κB in endothelial cells importantly contributes to inflammation (Baldwin, 1996). Therefore, we assumed that *NR4A1* potentially participates in the MAPK signaling pathway and negatively regulates inflammation in AV fistula. Particularly, we found that the biological process in AV fistula with the lower expression of *NR4A1* was majorly relevant to inflammation, which is consistent with our assumption.

Based on the microarray data of GSE 39488 (Hashimoto et al., 2013), Hashimoto et al. had reported several significant biological processes and pathways. Those reported biological processes in AV fistula, such as biological developmental processes and glycosaminoglycan binding, were different from our enrichment results such as cell adhesion, signal transduction, inflammatory response, apoptotic process. Our result tends to be much more relevant to intimal hyperplasia. The major enriched pathways in their study are the TGF-beta signaling pathway and the cytokines and inflammatory response pathway, and they were similar to our results. Furthermore, their study absents some pathways contained in our results such as MAPK signaling, NOD-like signaling, ABC transporters, and cell cycle. This difference may be attributed to different approaches. The enrichment analysis of pathway in their study was performed just based on identified DEGs, but this method is not sensitive enough to detect the subtle differences between the expression of individual genes (Mootha et al., 2003). GSEA was developed to focus on the changes of expression in *a priori* defined gene sets and resolves the problem of the undetectable, small changes in the expression of single genes (Mootha et al., 2003). We combined the ordinary enrichment analysis of KEGG pathway with GSEA to screen significant pathways. Additionally, their study did not identify any gene crucial for regulating biological processes in the outflow tract of AV fistula comparing with the normal vein. The best advantage of our study was the identification of 10 hub genes that potentially participate in intimal hyperplasia and 2 core genes that belong to the MAPK pathway. With the analysis of the GSE39488 dataset, the study from Jie et al. (2020) already found one pathway, i.e., NF-κB, and two hub genes that belong to NF-κB, and it is good for exploring the therapeutic management of AV fistula. However, the molecular mechanism of intimal hyperplasia varies; our study tries to explore the potential molecular with the same dataset (i.e., GSE39488) by a different procedure and cutoff value and could provide a new insight into future experimental validation. In addition, the cutoff value in the study by Jie et al. (2020) is too strict, and only 45 selected DEGs were used for the establishment of PPI networks resulting in a selection bias of the identification of hub gene. Furthermore, GSEA between the higher and lower expression of the *DUSP1* and *NR4A1* in our study helps us know what biological processes and pathways the two crucial genes might regulate, but the study from Jie et al. (2020) lacks this procedure.

Our study provided more information about the alteration of gene expression and biological processes in AV fistula. Future studies will try to validate whether the activation of *DUSP1* could attenuate cell proliferation, whether *NR4A1* possesses the ability to downregulate the inflammation level to inhibit intimal hyperplasia *in vitro* and *in vivo*, and how to participate in the activity of MAPK signaling pathway. However, some limitations were still presented in this bioinformatics study. First, although bioinformatics analysis was useful for an initial screen of crucial genes that participate in intimal hyperplasia, we cannot get any valid conclusion before sufficient validation. Moreover, how the crucial genes *DUSP1* and *NR4A1* to participate in the MAPK signaling pathway had not been verified in an experimental model to recognize the real activity in AV fistula. Second, the sample size of this dataset was relatively small. The acquisition of venous tissue after fistula is really difficult. The clinician could obtain the venous tissue of AV fistula only when this AV fistula is abandoned or abnormal and the patient has the opportunity to create the next new AV fistula at the ipsilateral limb. This dataset (i.e., GSE39488) was the biggest microarray data focused on human AV fistula tissue in the GEO database. Although the identification of DEGs and GSEA could not reflect the real activity in overflow AV fistula, this study could still provide the knowledge of gene expression in intimal hyperplasia under the circumstance lacking adequate evidence in humans, particularly in patients with uremia. Much more gene expression analysis studies with large sample sizes in humans are urgent to provide more strong evidence. Besides, experimental validation is required to know the potential role of *DUSP1* and *NR4A1* in intimal hyperplasia.

## Conclusion

The molecular mechanism of intimal hyperplasia resulting in venous vascular remodeling in AV fistula remains unclear. Our comprehensive bioinformatics analysis showed that the DEGs identified between venous tissues from AV fistula exposed to high blood flow with normal cephalic play a significant role in intimal hyperplasia of AV fistula. We identified 185 DEGs and screened four potential signaling pathways relevant to intimal hyperplasia such as MAPK signaling, Cell Cycle, NOD-like signaling, and TGF-beta signaling. A total of 10 hub genes were identified, and they were relevant to the regulation of intimal hyperplasia. Particularly, *DUSP1* and *NR4A1* might potentially participate in cell proliferation and inflammation, respectively, by the MAPK signaling pathway, and they serve as the therapeutic targets of intimal hyperplasia to prevent stenosis after AV fistula creation; however, they need further experimental validation.

## Data Availability Statement

The original contributions presented in the study are included in the article/Supplementary Material, further inquiries can be directed to the corresponding author/s.

## Author Contributions

ZZ mainly accomplished the project and drafted the original manuscript. QF provided the technical support and a critical review of the manuscript. LH partly participated in the study. YL led this study, made a contribution to the design of this project, and critically reviewed the manuscript. All authors contributed to the article and approved the submitted version.

## Conflict of Interest

The authors declare that the research was conducted in the absence of any commercial or financial relationships that could be construed as a potential conflict of interest.

## Publisher's Note

All claims expressed in this article are solely those of the authors and do not necessarily represent those of their affiliated organizations, or those of the publisher, the editors and the reviewers. Any product that may be evaluated in this article, or claim that may be made by its manufacturer, is not guaranteed or endorsed by the publisher.

## Abbreviations

eNOS: Endothelial NO synthase
TNF-α: Tumor necrosis factor alpha-α
IL-6: Interleukin 6
IL-8: Interleukin 8
IL-10: Interleukin 10
MMP-2: Matrix metallopeptidase 2
MMP-9: Matrix metallopeptidase 9
TIMP: Tissue inhibitor of metalloproteases
ADAMTS-1: ADAM metallopeptidase with thrombospondin type 1 motif 1
IGF-1: Insulin-like growth factor 1
PDGF: Platelet-derived growth factor
VEGF: Vascular endothelial-derived growth factor
VCAM-1: Vascular cell adhesion molecule-1
*EGR1*: Early growth response protein 1
*EGR2*: Early growth response protein 2
*EGR3*: Early growth response protein 3
*EGR4*: Early growth response protein 4
MKP-1: Mitogen-activated protein kinase phosphatase 1
*NR4A1*: Nuclear receptor subfamily 4 group A member 1
*NR4A2*: Nuclear receptor subfamily 4 group A member 2
*ATF3*: Activating Transcription Factor 3
*CXCR4*: C-X-C chemokine receptor type 4
*CCL4*: C–C Motif Chemokine Ligand 4
*CYR61*: Cysteine-rich angiogenic inducer 61
*DUSP1*: Dual specificity phosphatase 1
MAPK: Mitogen-activated protein kinase
NOD: Nucleotide-binding oligomerization domain
TGF-beta: Transforming growth factor-β
ERK: Extracellular-signal-regulated kinase
JNK: c-Jun N-terminal kinase
NLRP3: NOD-, LRR-, and pyrin domain-containing protein 3.
