# Supplementary material for: Identification of the Crucial Gene in Overflow Arteriovenous Fistula by Bioinformatics Analysis
Source: Front Physiol. 2021 Aug 4;12:621830. doi: 10.3389/fphys.2021.621830 (PMC8371383; doi:10.3389/fphys.2021.621830)

Supplementary Figure 1. The Venn diagram between hub gene and four crucial gene set a. MAPK signaling pathway; b. NOD-like signaling pathway; c. Cell cycle; d. TGF-beta signaling pathway.

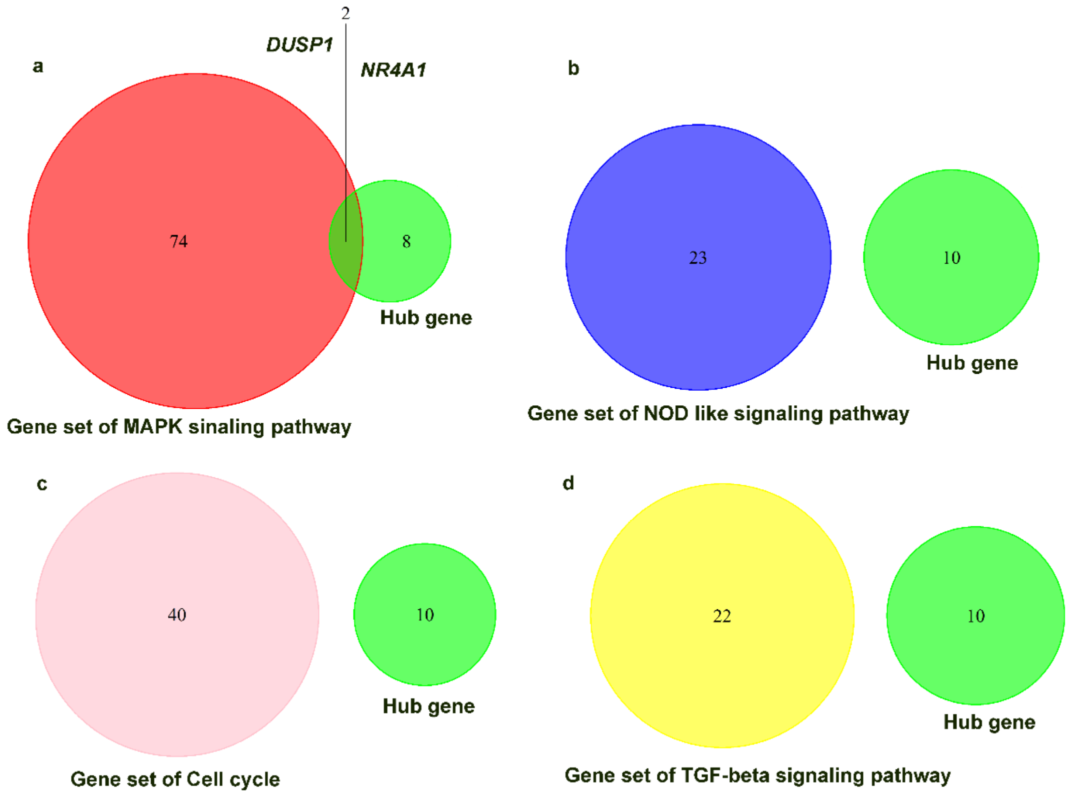

Supplementary Figure 2. The Boxplot of Expression Value of Identified Hub Gene Comparing Arteriovenous fistula With Control.

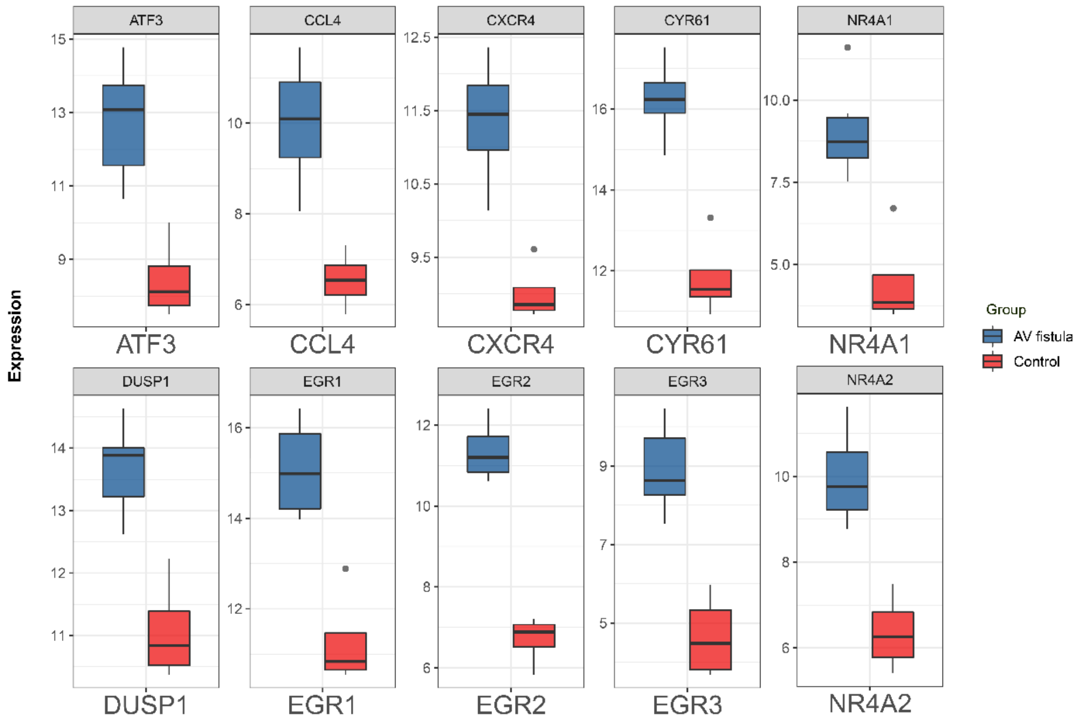

Supplement: Supplementary file 2 [file Data_Sheet_1.PDF]
